# Periplocin Targets HDAC10 to Inhibit NF-κB Signaling and Induce Apoptosis in Myeloid Leukemia Cells

**DOI:** 10.7150/jca.113591

**Published:** 2025-06-23

**Authors:** Wenjie Li, Shuping Lai, Jingxian Chen, Ziang Chen, Yanying Zhou, Rongfang Wei, Yan Chen

**Affiliations:** Department of Hematology, The Eighth Affiliated Hospital, Sun Yat-sen University, 3025 Shennan Middle Road, Shenzhen 518033, China.

**Keywords:** leukemia, epigenetics, HDAC10, periplocin, NF-κB

## Abstract

**Background**: Periplocin, a bioactive compound extracted from *Cortex periplocae*, has long been employed in traditional medicine for its diverse therapeutic effects, particularly in alleviating inflammation and inhibiting cancer progression. However, despite its potential benefits, the underlying molecular mechanisms of periplocin, especially in the context of leukemia treatment, remain poorly elucidated, warranting further investigation to uncover its precise role and therapeutic targets.

**Methods**: A comprehensive approach combining network pharmacology and transcriptomic analysis was utilized to identify HDAC10 as a critical downstream target of periplocin. Molecular docking and dynamic simulation studies were performed to elucidate the interaction between periplocin and HDAC10 at the molecular level. Additionally, functional assays, including apoptosis induction, cell cycle regulation, and pathway inhibition experiments, were conducted to validate the mechanistic role of HDAC10 and its relevance to periplocin's anti-leukemic effects.

**Results**: Periplocin was identified as an effective inhibitor of HDAC10, binding specifically to its hydrophobic active pocket and suppressing its enzymatic activity. This inhibition disrupted downstream signaling, particularly the NF-κB pathway, leading to significant apoptosis and cell cycle arrest in leukemia cells. These results therapy, offering insights into its mechanism of action through HDAC10 targeting.

**Conclusion**: In conclusion, periplocin, as a novel natural compound, exhibits significant anti-leukemia activity, highlighting its potential as a promising therapeutic candidate for leukemia treatment. The findings contribute to the growing interest in natural compounds as innovative solutions for addressing unmet clinical needs in hematological malignancies.

## 1. Introduction

Leukemia is a complex and aggressive blood cancer that arises from hematopoietic stem cells (HSCs), characterized by its diverse subtypes and high variability in clinical presentation and progression. Its pathogenesis is complex and multifactorial, involving chromosomal translocations, oncogene activation, tumor suppressor gene inactivation, transcription factor dysfunction, aberrant growth factor signaling, and dysregulated pathways 1-3. These abnormalities disrupt normal hematopoietic stem cell differentiation or lead to aberrant differentiation, resulting in the accumulation of leukemia stem cells (LSCs) 4-6. Intriguingly, a significant subset of leukemia patients exhibits no detectable chromosomal abnormalities or known gene mutations, suggesting that factors beyond traditional genetic mechanisms contribute to leukemogenesis 7.

Epigenetics has emerged as a pivotal mechanism underlying cancer biology. Epigenetics refers to heritable changes in gene expression that do not involve alterations to the underlying DNA sequence, including mechanisms such as DNA modifications (e.g., methylation and hydroxymethylation), histone modifications (e.g., acetylation, methylation, and phosphorylation), and RNA modifications (e.g., m6A methylation) 8 9. Increasing evidence highlights the role of epigenetic dysregulation in the initiation and progression of leukemia. For instance, mutations in DNMT3A and TET2 disrupt normal clonal hematopoiesis and are implicated in the transformation of HSCs into myelodysplastic syndromes (MDS) and acute myeloid leukemia (AML) 10-12. Similarly, leukemogenic fusion genes such as PML-RARA recruit histone deacetylases (HDACs) to specific promoters, leading to transcriptional silencing of tumor suppressor genes 13. Aberrant histone methylation mediated by MLL (KMT2A) translocations further underscores the central role of histone modifications in driving leukemia 14. Moreover, RNA epigenetics, such as dysregulated m6A modifications by factors like METTL3, have been shown to enhance the stability and translation of oncogenic transcripts, including MYC and BCL2, promoting leukemia cell proliferation and inhibiting apoptosis 15. These findings establish epigenetic alterations as critical contributors to leukemia pathogenesis and highlight their potential as therapeutic targets.

Histone acetylation, an essential epigenetic modification, influences chromatin structure and regulates gene expression by modifying lysine residues on histone tails. Acetylation promotes chromatin relaxation, facilitating transcription, while histone deacetylation, mediated by HDACs, compacts chromatin and represses gene expression 16-17. In leukemia, HDACs are frequently overexpressed and associated with poor prognosis. For example, HDAC1 and HDAC2 are highly expressed in AML and suppress the expression of tumor suppressor genes such as p21, contributing to disease progression 18-20. HDAC3, on the other hand, has been implicated in therapy resistance, making it a promising target for overcoming drug resistance and associated with leukemia stem cell 21-23. Consequently, HDAC inhibitors (HDACis) have emerged as an important class of epigenetic therapeutics. Clinically approved HDACis, such as romidepsin and panobinostat, have shown efficacy in treating hematologic malignancies, including multiple myeloma and peripheral T-cell lymphoma 24-25, 26. However, their application is constrained by notable side effects, including bone marrow suppression and cardiotoxicity, along with the development of drug resistance. These challenges highlight the pressing need for discovering new and safer HDAC inhibitors.

Derived from the medicinal plant Cortex periplocae, Periplocin represents a promising natural alternative to synthetic drugs, with demonstrated anticancer efficacy in a range of solid tumors, including breast cancer, lung cancer, and hepatocellular carcinoma 27-28. Previous studies have demonstrated that periplocin exerts its antitumor effects through multiple mechanisms, including the regulation of cell cycle-related proteins such as Cyclin D1 and CDK4, and the induction of apoptosis via the mitochondrial pathway 29-30. Furthermore, periplocin has been shown to inhibit critical oncogenic pathways, such as NF-κB and PI3K-AKT, thereby suppressing cancer cell proliferation, invasion, and metastasis 30. These findings highlight its broad anticancer potential and provide a strong rationale for exploring its role in hematologic malignancies. However, despite these promising results, the therapeutic potential of periplocin in leukemia remains uninvestigated, and its molecular targets and mechanisms of action in this context have yet to be elucidated.

In this study, we utilized network pharmacology, a systems biology approach that combines computational predictions with experimental validation 31, to investigate the antileukemic potential of periplocin. By identifying drug targets and mapping multi-target, multi-pathway interactions, network pharmacology offers a powerful framework for dissecting the mechanisms of action in complex diseases like leukemia. Our findings reveal that periplocin exerts significant antileukemic effects, particularly in AML, with HDAC10 emerging as a key molecular target identified through network pharmacology analysis.

Molecular docking serves as a critical tool for investigating ligand-protein interactions and pinpointing therapeutic targets with high precision 32-33. Leveraging advancements in protein structure prediction, particularly AlphaFold3, we accurately modeled the three-dimensional structure of HDAC10. This structure was integrated with Schrödinger's docking tools to simulate the binding dynamics of periplocin to HDAC10's active hydrophobic pocket. The results shed light on the molecular basis of periplocin's selective targeting of HDAC10, offering mechanistic insights into its epigenetic modulation in AML. Furthermore, these findings provide a scientific framework for future HDAC10-targeted drug development.

## 2. Methods and Materials

### 2.1 Cell culture

Human monocytic AML cells (THP-1) were generously provided by the He Lab. THP-1 and K562 cells (ATCC, CCL-243) were cultured in Roswell Park Memorial Institute (RPMI) 1640 medium (Corning, 10-040-CV) supplemented with 10% fetal bovine serum (FBS, ExCell Bio, FNA500). Cells were maintained at 37°C in a humidified atmosphere containing 5% CO₂ under normoxic conditions.

### 2.2 CCK-8 assay for cell proliferation and determination of periplocin IC50

Cell proliferation was assessed with the Cell Counting Kit-8 (CCK-8) assay following the manufacturer's instructions. Cells were seeded in 96-well plates at 5000 cells per well and incubated overnight at 37°C with 5% CO₂. To determine the half-maximal inhibitory concentration (IC_50_), cells were exposed to various concentrations of Periplocin (MCE, HY-N1381). After 48 or 72 hours, 10 µL of CCK-8 reagent was added to each well, and the plates were incubated for 2 hours at 37°C. The absorbance was measured at 450 nm using a microplate reader. IC_50_ values, defined as the Periplocin concentration that inhibits 50% of cell viability, were calculated through nonlinear regression analysis. All experiments were conducted in triplicate, with results expressed as mean ± standard deviation.

### 2.3 Annexin V/PI flow cytometry for apoptosis detection

Apoptosis analysis was performed using the Annexin V-FITC/PI apoptosis detection kit (Elabscience, E-CK-A217) according to the provided instructions. After treatment, cells were collected, rinsed twice with chilled phosphate-buffered saline (PBS), and resuspended in 1× binding buffer at a final concentration of 5 × 10⁵ cells/mL. Subsequently, 2.5 µL of Annexin V-FITC and 2.5 µL of propidium iodide (PI) solution were added to 100 µL of the cell suspension. The mixture was gently vortexed and incubated in the dark at room temperature for 20 minutes. After staining, cells were analyzed using flow cytometry (BD FACSCanto II) within one hour. Data acquisition and analysis were performed using Flowjo_v10.8.1.

### 2.4 BrdU incorporation assay for cell proliferation

Cell proliferation was evaluated using the BrdU incorporation assay with anti-BrdU antibodies (BioLegend,364113) and 7-AAD (Biolegend,420403) for total DNA staining. Cells were incubated with 10 µM BrdU (BioLegend,423401) for 1 hour before harvesting. Following incubation, cells were fixed and permeabilized using Cyto-Fast™ Fix/Perm Buffer Set (Biolegend,426803) according to the manufacturer's instructions. Cells were then incubated with an anti-BrdU antibody (BioLegend, 364113) in the dark for 30 minutes at room temperature. After washing, cells were resuspended in staining buffer containing 7-AAD for DNA content analysis. Samples were analyzed by flow cytometry, with BrdU-positive cells indicating proliferative activity and 7-AAD providing total DNA content information. Data were analyzed using appropriate software to assess cell cycle distribution and proliferation status.

### 2.5 Total RNA extraction and quantitative PCR (qPCR)

K562 and THP-1 cells from both experimental (treated with 100 nM periplocin) and control (treated with DMSO) groups were collected after 72 hours of treatment. The cells were washed once with pre-chilled PBS and centrifuged at 300 g for 5 minutes at 4°C to remove residual medium. Subsequently, 1 mL of TRIzol reagent (Thermo Fisher Scientific,15596018CN) was added per 1 million cells for cell lysis, and total RNA was extracted following the manufacturer's protocol.

For cDNA synthesis, 1 µg of total RNA from each sample was reverse-transcribed using a commercially available reverse transcription kit (Vazyme, R312-01), according to the kit instructions. Real-time quantitative PCR (qPCR) was performed using SYBR Green PCR Master Mix (Vazyme, Q711-02-AA) on a qPCR system. Human GAPDH was used as the internal control to normalize RNA input. Each qPCR reaction was conducted in a total volume of 10 µL as recommended by the manufacturer.

Primer sequences used for qPCR are as follows: HDAC10 forward primer: ATCTCTTTGAGGATGACCCCAG; HDAC10 reverse primer: ACTGCGTCTGCATCTGACTCTC.

GAPDH forward primer: TGCACCACCAACTGCTTAG; GAPDH reverse primer: AGTAGAGGCAGGGATGATGTTC. The relative gene expression levels were calculated using the 2^-ΔΔCt method, and all reactions were performed in triplicate to ensure reproducibility.

### 2.6 Protein extraction and western blot analysis

Cells were collected and rinsed twice with cold phosphate-buffered saline (PBS). The resulting cell pellets were centrifuged at 4 °C and then resuspended in pre-chilled lysis buffer (Affinibody, AIWB-012), followed by gentle pipetting to ensure thorough mixing. Samples were lysed on ice for 20 minutes until no visible cell pellet remained. The lysates were subjected to centrifugation at 10,000g for 10 minutes at 4°C, after which the supernatants were carefully transferred to fresh microcentrifuge tubes. Protein concentrations were measured using the Pierce BCA Protein Assay Kit (Thermo Fisher Scientific, 23225) following the provided protocol. Equal amounts of protein were mixed with 5X loading buffer and boiled for 10 minutes.

Proteins were separated on SDS-PAGE gels (ACE Biotechnology, ET12412Gel) and transferred onto nitrocellulose membranes (Bio-Rad, 1620112). The membranes were blocked and then incubated with primary antibodies against p65 (CST, 8242), phosphorylated p65 (p-p65) (UpingBio technology, YP-Ab-01272), HDAC10 (UpingBio technology, YP-Ab-01781), and β-actin (Proteintech, 66009-1-Ig). The membranes were washed and treated with the corresponding secondary antibodies (IRDye® 680RD Goat anti-Mouse IgG Secondary Antibody, 926-68070; IRDye® 800CW Goat anti-Rabbit IgG Secondary Antibody, 926-32211). Protein bands were visualized using the Odyssey imaging system, and data were analyzed accordingly.

### 2.7 RNA sequencing analysis

K562 and THP-1 cells were treated with DMSO (control group) or 100 nM Periplocin (experimental group) for 72 hours. At the end of the 72-hour treatment period, cells were harvested and washed once with pre-chilled PBS (4 °C, 300 × g, 5 minutes) to remove any residual medium. Subsequently, each sample (1 × 10⁶ cells) was lysed in 1 mL of TRIzol reagent to ensure complete RNA extraction. The lysates were immediately stored at -80 °C for downstream RNA analysis. Each condition was analyzed using a single biological replicate. The RNA-seq data were used as an exploratory tool to identify potential signaling pathways, which were subsequently validated through independent experiments.

Total RNA was extracted and purified according to the TRIzol reagent manufacturer's instructions. The RNA purity and concentration were assessed using a spectrophotometer, and RNA integrity was evaluated using a Bioanalyzer. Samples with RNA integrity number (RIN) ≥7 were deemed suitable for RNA sequencing.

RNA sequencing was conducted by Gene Denovo Biotechnology Co. (Guangzhou, China). Library preparation was performed using poly-A tail enrichment to isolate mRNA, followed by the construction of paired-end sequencing libraries. Sequencing was performed on the T7 platform, generating 150 bp paired-end reads. Initial raw data underwent quality control using the company's standard pipeline to produce clean, high-quality reads for downstream bioinformatics analyses.

### 2.8 Construction of active compound-target network

Cytoscape is a powerful tool for visualizing network pharmacology and analyzing interactions. Utilizing Cytoscape 3.10.0, the active compounds of periplocin and their respective targets were imported, enabling the construction of a compound-target network specific to periplocin. This network consists of nodes representing either active compounds or their corresponding targets, with the connections between them depicted as edges. The network provides a comprehensive visualization of the interaction landscape of periplocin's active components and their associated targets.

### 2.9 Identification of predicted targets of leukemia

The potential targets associated with leukemia were identified using multiple databases, including Genecards (https://www.genecards.org/), DisGeNET (a comprehensive database of gene-disease associations), and OMIM (https://www.omim.org/). The keyword “leukemia” was used to search these databases, and relevant disease-related targets were extracted from each source. The targets obtained from Genecards and DisGeNET were filtered based on a relevance score threshold of 0.3 or above to ensure specificity. The results from all databases were merged, and duplicate entries were removed to create a non-redundant list of leukemia-related targets. These disease targets were subsequently integrated with periplocin's target network to identify overlapping nodes, representing potential therapeutic targets of periplocin in leukemia.

### 2.10 Identification of overlapping targets using a Venn diagram

To identify the common targets between periplocin and leukemia, the predicted targets of periplocin and the disease-related targets of leukemia were compared. The overlapping targets were visualized using a Venn diagram generated in R software (version 4.3.0) with the “VennDiagram” package. This approach allowed for a clear visualization of the unique and shared targets between the two datasets, facilitating further enrichment analysis.

### 2.11 Gene Ontology (GO) enrichment analysis

To uncover the biological significance of the overlapping targets, GO enrichment analysis was performed, focusing on three primary domains: biological processes (BP), cellular components (CC), and molecular functions (MF). The "clusterProfiler" package in R was employed for annotation, with a threshold of p < 0.05 to ensure statistical significance. Key enriched GO terms in each category were visualized using bar charts, highlighting the most relevant biological functions associated with the shared targets. This analysis provided insights into the molecular mechanisms underlying the targets' roles within the context of this study.

### 2.12 KEGG pathway enrichment analysis

To explore the potential pathways underlying the therapeutic effects of periplocin in leukemia, KEGG pathway enrichment analysis was performed. Using the “clusterProfiler” package in R, the overlapping targets were mapped to KEGG pathways, with pathways achieving a significance threshold of p < 0.05 considered enriched. The results were presented as bubble plots, where bubble size corresponded to the number of genes involved in each pathway, and color reflected the significance level (-log10(p-value)). This analysis revealed key signaling pathways potentially modulated by periplocin, providing valuable insights into its mechanism of action in leukemia.

### 2.13 Molecular docking

The HDAC10 protein structure was predicted using AlphaFold3. The obtained protein crystal structure was processed using the Protein Preparation Wizard module in the Schrödinger software. The preprocessing steps included assigning bond orders, optimizing H-bond assignments, regenerating states of native ligands, minimizing the protein energy, and removing water molecules. The 2D structure of periplocin in SDF format was processed using the LigPrep module in Schrödinger to generate its 3D conformations. The generated conformers were optimized for docking. Potential binding sites on the HDAC10 protein were predicted using the SiteMap module in Schrödinger. The best binding site was selected, and an enclosing box was generated around this site using the Receptor Grid Generation module. This step defined the active binding site of HDAC10 for subsequent docking. The processed ligand (periplocin) was docked into the identified active binding site of HDAC10 using the extra precision (XP) docking mode in Schrödinger's Glide module. Docking scores were calculated to evaluate the binding affinity, with lower docking scores indicating stronger and more stable interactions between the ligand and the protein. The binding affinity of the periplocin-HDAC10 complex was further refined using MM-GBSA (Molecular Mechanics-Generalized Born Surface Area) calculations. The ΔG binding energy (dG Bind) was computed to represent the free energy of binding. A lower ΔG value corresponds to a more stable ligand-protein interaction.

### 2.14 Plasmid construction and knockdown cell line construction

The plko.1 vector was used as the backbone for constructing human HDAC10 shRNA plasmids. The target sequences for human HDAC10 shRNA were as follows:

1. CAGGTGAACAGTGGTATAGCA

2. CGGGTTCTGTGTGTTCAACAA

Lentiviral particles encoding shRNA targeting HDAC10 were generated using a lentiviral packaging system according to standard protocols. K562 and THP-1 cells were infected with the lentiviral particles. After 48 hours, transduced cells were subjected to puromycin selection (2 μg/mL for K562; 1 μg/mL for THP-1) to establish stable HDAC10 knockdown cell lines. Knockdown efficiency was confirmed by western blot analysis.

## 3. Results

### 3.1 Periplocin induces cell cycle arrest and induction of apoptosis

**Figure 1:**
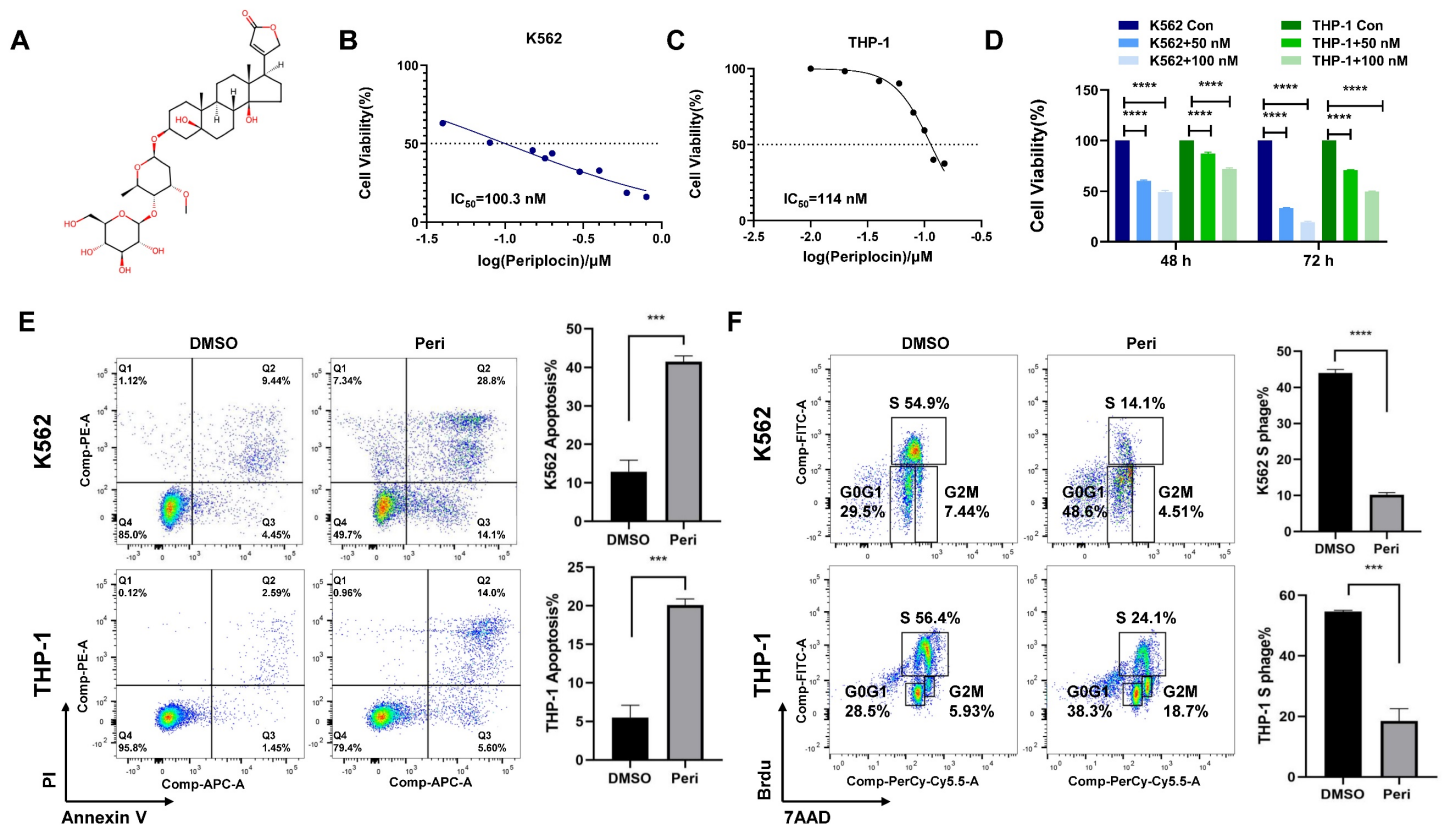
** Periplocin inhibits proliferation, induces apoptosis, and causes cell cycle arrest in leukemia cells.** (A) Chemical structure of periplocin. (B-C) IC₅₀ values of Periplocin in K562 and THP-1 cells were 100.3 nM and 114 nM, respectively. (D) Proliferation of K562 and THP-1 cells after 48 h and 72 h treatment with different concentrations of Periplocin. (E) Flow cytometry analysis of apoptosis in K562 and THP-1 cells treated with 100 nM Periplocin for 72 h. (F) Cell cycle changes in K562 and THP-1 cells treated with 100 nM Periplocin for 72 h. Data are presented as mean ± SD. ***p < 0.001; ****p < 0.0001.

Consistent with previous studies highlighting the anti-cancer potential of natural compounds, we investigated the effects of Periplocin, whose chemical structure is shown in **Figure 1A**. IC_50_ values determined from dose-response curves demonstrated that Periplocin exerts a concentration-dependent cytotoxic effect, with IC_50_ values of approximately 100 nM in K562 cells and 110 nM in THP-1 cells (**Figure 1B-C**). Proliferation assays further revealed that treatment with 50 nM and 100 nM Periplocin for 48 and 72 hours significantly reduced cell viability in both cell lines compared to controls, with reductions of up to 40-50% at 100 nM after 72 hours (**Figure 1D**, p < 0.0001). Flow cytometry analysis of Annexin V/PI staining showed that periplocin treatment (100 nM for 72 hours) significantly increased the proportion of apoptotic cells. Specifically, the percentage of early and late apoptotic cells increased from 9.92% to 42.9% in K562 cells and from 4.04% to 19.6% in THP-1 cells (**Figure 1E**). Additionally, periplocin induces a robust G0/G1 phase arrest and S phase reduction, which likely contributes to increase in apoptosis. The G0/G1 population increased from 29% to 48.5% in K562 cells, while the S phase decreased from 54.9% to 14.1%. Similarly, the G0/G1 phase increased from 28.5% to 38.3%, with a reduction in the S phase from 56.4% to 24.1% in THP-1 cells (**Figure 1F**). These findings collectively highlight the potential of Periplocin as a therapeutic agent targeting leukemia cell growth through apoptosis induction and cell cycle blockade.

### 3.2 Transcriptome profiling and pathway enrichment reveal mechanistic insights into periplocin's effects on leukemia cells

**Figure 2:**
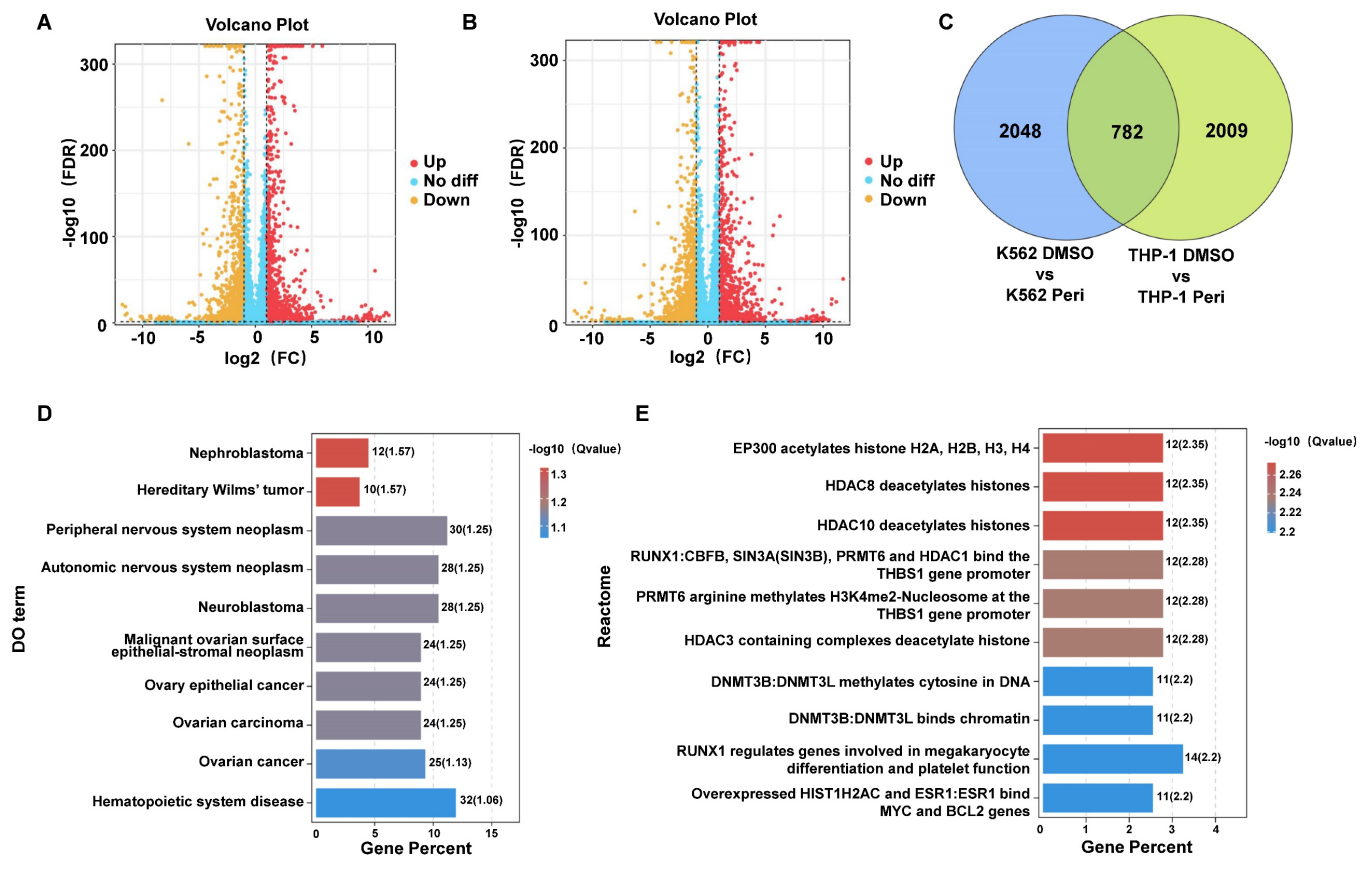
** Transcriptomic Analysis Reveals Differential Gene Expression and Pathway Enrichment in Periplocin-Treated Leukemia Cells.** (A-B) Differentially expressed genes identified through transcriptome sequencing in K562 cells (left panel) and THP-1 cells (right panel). (C) Venn diagram illustrating the intersection of differentially expressed genes between the two groups. (D) Bar plot showing pathway enrichment from DO (Disease Ontology) analysis of the identified genes. (E)Bar plot showing pathway enrichment from Reactome pathway analysis.

Transcriptome sequencing of K562 and THP-1 cells treated with Periplocin revealed significant transcriptional changes. Volcano plots of differentially expressed genes (DEGs) in K562 and THP-1 cells show distinct patterns of gene upregulation and downregulation (**Figure 2A-B**). Venn diagram analysis demonstrated 782 shared DEGs between K562 and THP-1 cells, with 2048 and 2009 unique DEGs specific to K562 and THP-1 cells, respectively, indicating both common and cell line-specific responses to Periplocin treatment (**Figure 2C**). Pathway enrichment using Disease Ontology (DO) terms highlighted associations with hematopoietic system diseases, neuroblastoma, ovarian cancer, and other cancer-related pathways, suggesting Periplocin's broad regulatory effects on cancer-related pathways (**Figure 2D**). Reactome pathway analysis further underscored the involvement of epigenetic regulation and chromatin remodeling processes, providing insights into Periplocin's role as a modulator of leukemia cell epigenetic landscapes (**Figure 2E**).

**Figure 3:**
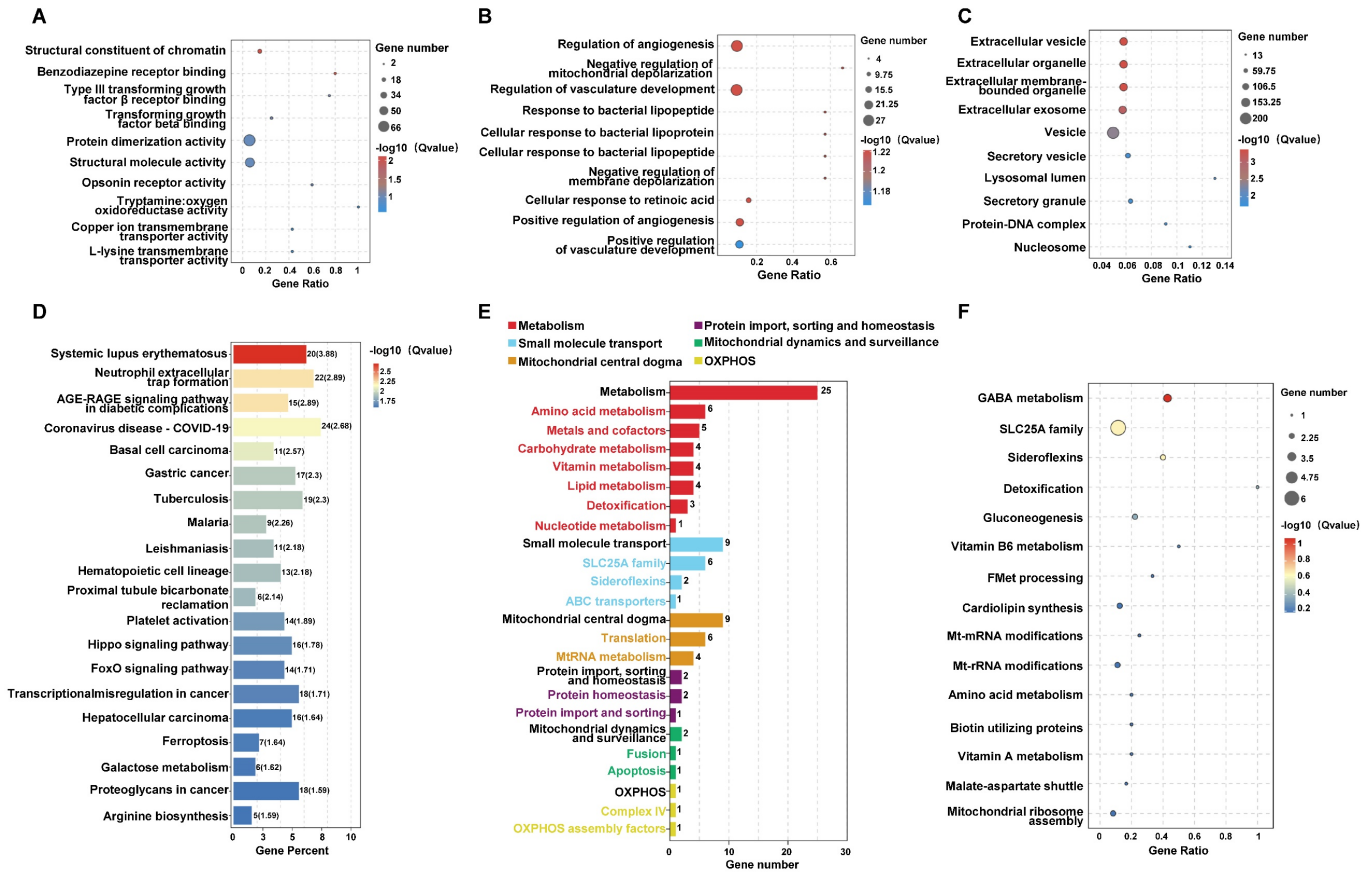
** Pathway and functional enrichment analysis of genes affected by periplocin in leukemia cells.** (A-C) Bubble plot depicting pathway enrichment from GO (Gene Ontology) analysis of the identified genes. (A) GO analysis results for Biological Process (BP) category. (B) GO analysis results for Molecular Function (MF) category. (C) GO analysis results for Cellular Component (CC) category. (D) Bar plot depicting pathway enrichment from KEGG (Kyoto Encyclopedia of Genes and Genomes) pathway analysis. (E) Mito-pathway bar plot showing the number of genes associated with various mitochondrial metabolic processes and functions, including metabolism, small molecule transport, mitochondrial central dogma, protein import, sorting and homeostasis, mitochondrial dynamics and surveillance, and OXPHOS (oxidative phosphorylation) assembly factors. The number of genes involved in each category is displayed beside the respective bar. (F) Bubble plot depicting pathway enrichment in mitochondrial pathways, including GABA metabolism, SLC25A family, sideroflexins, detoxification, and other metabolic processes. The size of each bubble corresponds to the number of genes, while the color gradient indicates the -log10 (q value) for the enrichment significance.

Gene ontology (GO) enrichment analysis was further performed to classify the DEGs according to their biological roles. The GO analysis results for Biological Process (BP) revealed significant enrichment in pathways related to angiogenesis, mitochondrial depolarization regulation, and cellular responses to bacterial lipoprotein (**Figure 3A**). Molecular Function (MF) analysis highlighted terms such as structural constituent of chromatin and receptor binding (**Figure 3B**), while Cellular Component (CC) analysis emphasized extracellular vesicles, exosomes, and other membrane-bounded organelles (**Figure 3C**). Pathway enrichment analysis using the Kyoto Encyclopedia of Genes and Genomes (KEGG) highlighted pathways including hematopoietic cell lineage and various metabolic and signaling processes relevant to cancer (**Figure 3D**). These comprehensive analyses suggest that Periplocin exerts multifaceted effects on leukemia cells by modulating critical cellular pathways and processes.

### 3.3 Mitochondrial pathway analysis highlights metabolic alterations induced by periplocin treatment

Cell metabolism is critical for tumor survival, as reflected in our sequencing data. Analysis of mitochondria-related pathways revealed significant enrichment in metabolic pathways (**Figure 3E**). Bubble plots also highlighted notable differences in pathways such as detoxification, GABA metabolism, gluconeogenesis, and cardiolipin synthesis. Additionally, other mitochondria-associated pathways, such as small molecule transport, were significantly enriched, particularly involving the SLC25A family (**Figure 3F**). Taken together, these findings suggest that periplocin may exert its antitumor effects, to some extent, by influencing tumor cell metabolism.

### 3.4 Network pharmacology analysis highlights common targets and pathway enrichment for periplocin in leukemia

**Figure 4:**
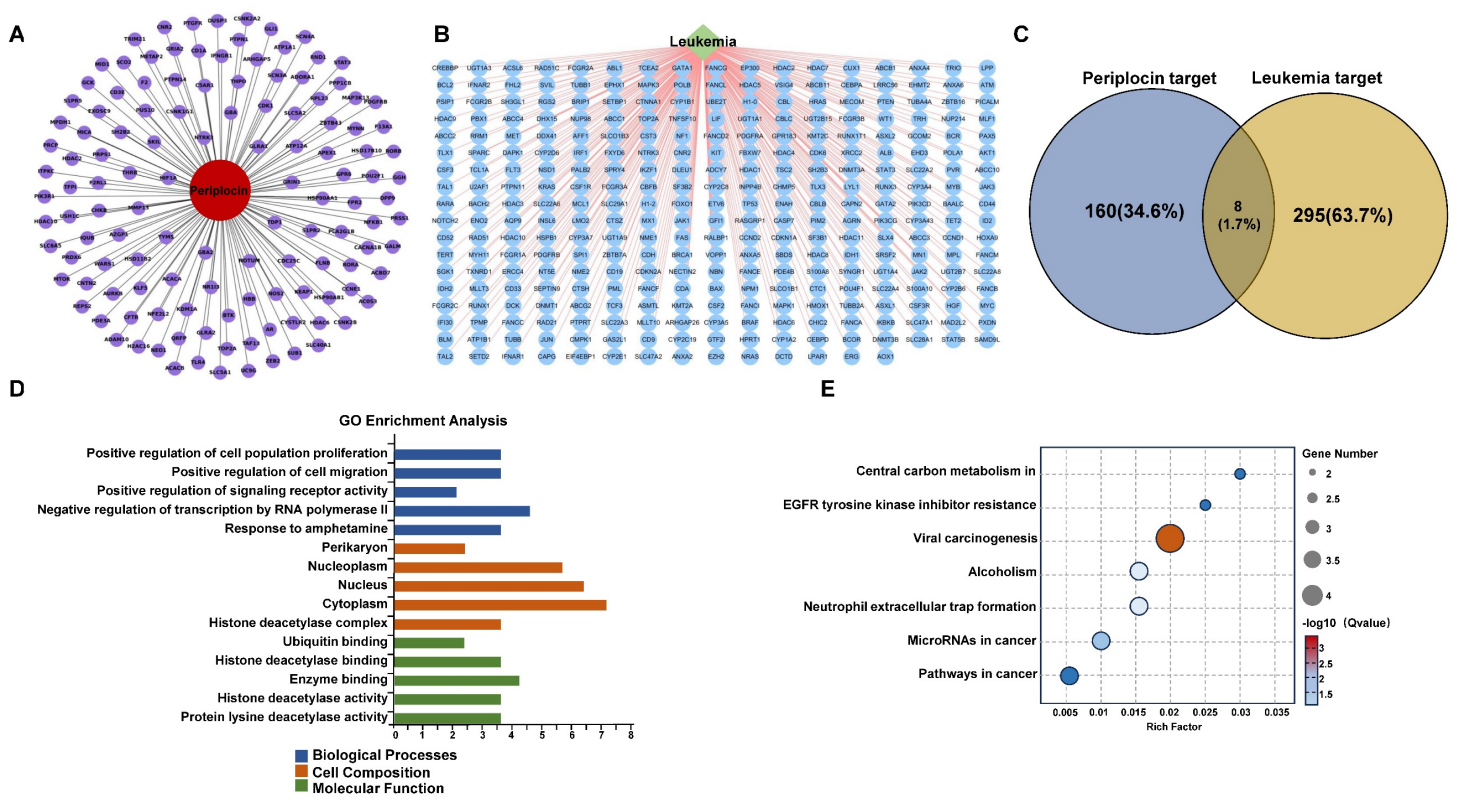
** Drug-target interaction network and functional enrichment analysis of periplocin in leukemia.** (A) Drug-target interaction network for Periplocin (nodes represent genes associated with the drug, and edges represent interactions between the drug and the targets). (B) Leukemia-related targets. (C) Venn diagram showing the intersection of Periplocin targets and leukemia-related targets. (D-E) GO pathway enrichment bar chart (D) and KEGG pathway enrichment bubble plot (E).

To better understand the connection between Periplocin and leukemia, we employed a network pharmacology approach to identify overlapping targets. This analysis revealed 8 shared genes between Periplocin's predicted targets and leukemia-associated genes (**Figure 4A-C**). GO and KEGG enrichment analyses of these common targets indicated significant involvement in cancer-related pathways. Notably, histone acetylation-related pathways were enriched in the molecular function category of the GO analysis, closely aligning with the earlier transcriptomic findings (**Figure 4D-E**). This evidence implies that Periplocin's therapeutic effects may be mediated through modulation of pathways associated with epigenetic regulation that critical to tumor progression.

### 3.5 Identification and validation of core targets of periplocin in leukemia using PPI network and clinical data analysis

**Figure 5:**
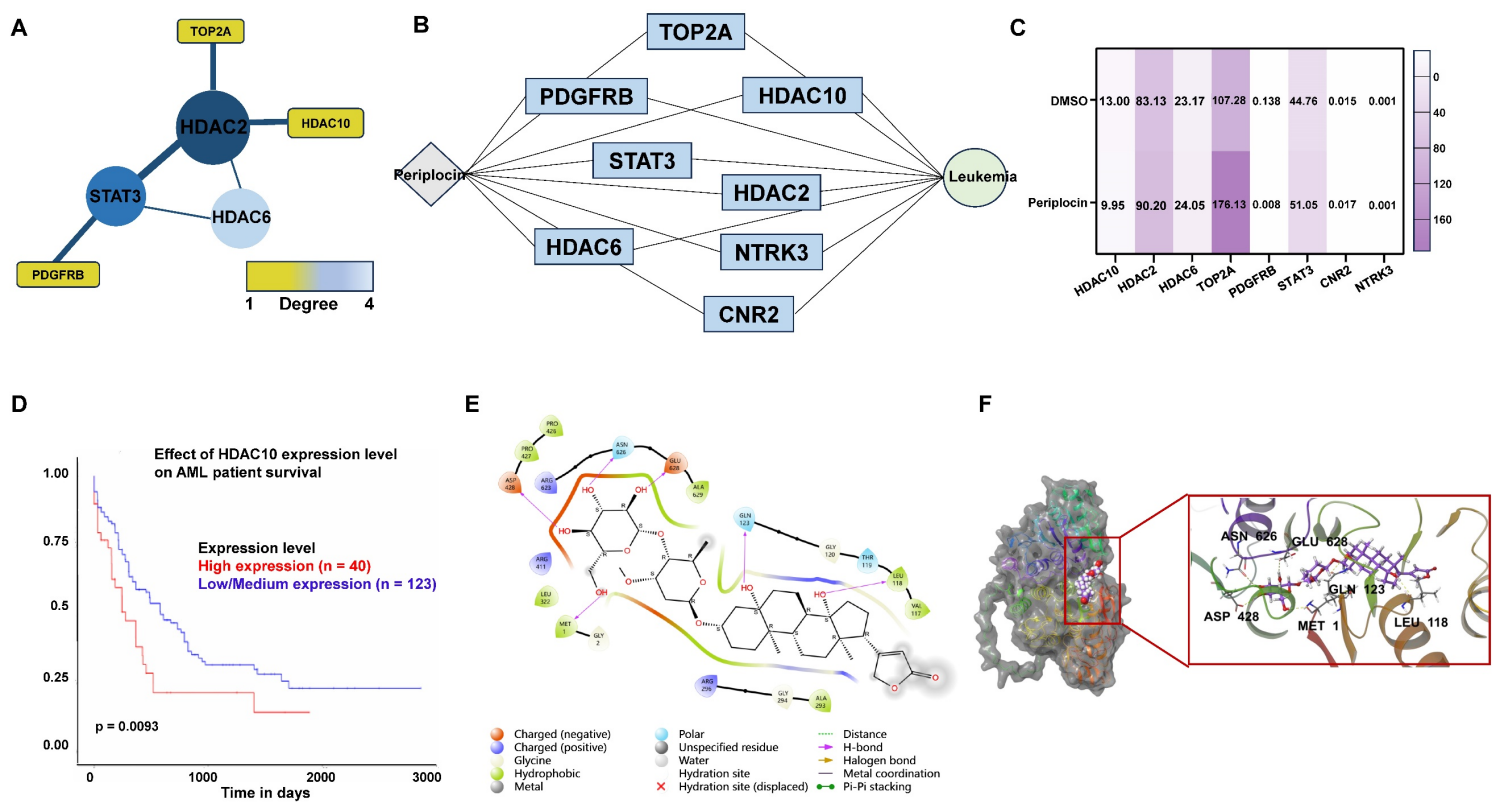
** Periplocin-target interaction network, hdac10 expression, and molecular docking in leukemia.** (A) Protein-protein interaction (PPI) network of intersecting genes targeted by periplocin and their relation to leukemia. (B) Interaction network depicting the relationship between periplocin, its targets, and leukemia-related factors. (C) Heatmap illustrating the expression levels of intersecting target genes from transcriptome sequencing data. (D) Kaplan-Meier (KM) survival curve showing the association between HDAC10 expression and prognosis in the TCGA database. (E-F) Molecular docking diagram showing the interaction between Periplocin and HDAC10, highlighting hydrophobic interactions with ALA629 and hydrogen bonds with residues LEU118, GLN123, GLU628, ASN626, ASP428, and MET1 in the active pocket of HDAC10.

To identify the target molecules of Periplocin among the overlapping genes, we constructed a PPI network (**Figure 5A**) and a Periplocin-leukemia shared target network (**Figure 5B**). Notably, histone deacetylation-related molecules, such as HDAC2, HDAC6, and HDAC10, accounted for a significant proportion within these networks. Transcriptome sequencing data were analyzed for differentially expressed genes. Among the overlapping genes, HDAC10 emerged as the most significant, showing marked downregulation in response to Periplocin treatment (**Figure 5C**). Given this observation, we further investigated the clinical relevance of HDAC10 using prognosis data from the TCGA database. The analysis revealed that high HDAC10 expression is significantly associated with poorer patient outcomes (p = 0.0093), underscoring its potential role in promoting tumor progression and its value as a therapeutic target (**Figure 5D**). To evaluate the safety profile of targeting HDAC10 in disease treatment, we analyzed its expression using the BloodSpot database (https://www.fobinf.com/) (**Supplementary Figure 1A**). The results revealed that HDAC10 expression in hematopoietic stem cells (HSCs) and early progenitor populations (e.g., GMP, CMP, MEP) is significantly lower than that observed in various acute myeloid leukemia subtypes.

To better understand the molecular basis of the interaction between Periplocin and HDAC10, molecular docking analysis was performed. The results demonstrated that Periplocin binds specifically to the active hydrophobic pocket of HDAC10, forming stable interactions, including hydrophobic binding with ALA629 and hydrogen bonds with critical residues such as LEU118, GLN123, GLU628, ASN626, ASP428, and MET1 (**Figure 5E**). Three-dimensional structural visualization confirmed that Periplocin fits securely within the active pocket, reinforcing the stability of these interactions (**Figure 5F**). The molecular docking results further suggest that Periplocin can directly interact with HDAC10. Combined with the above findings, this supports the accuracy of HDAC10 as a downstream target of Periplocin.

### 3.6 Periplocin induces apoptosis in leukemia cells by targeting HDAC10 and inhibiting the NF-κB pathway

**Figure 6:**
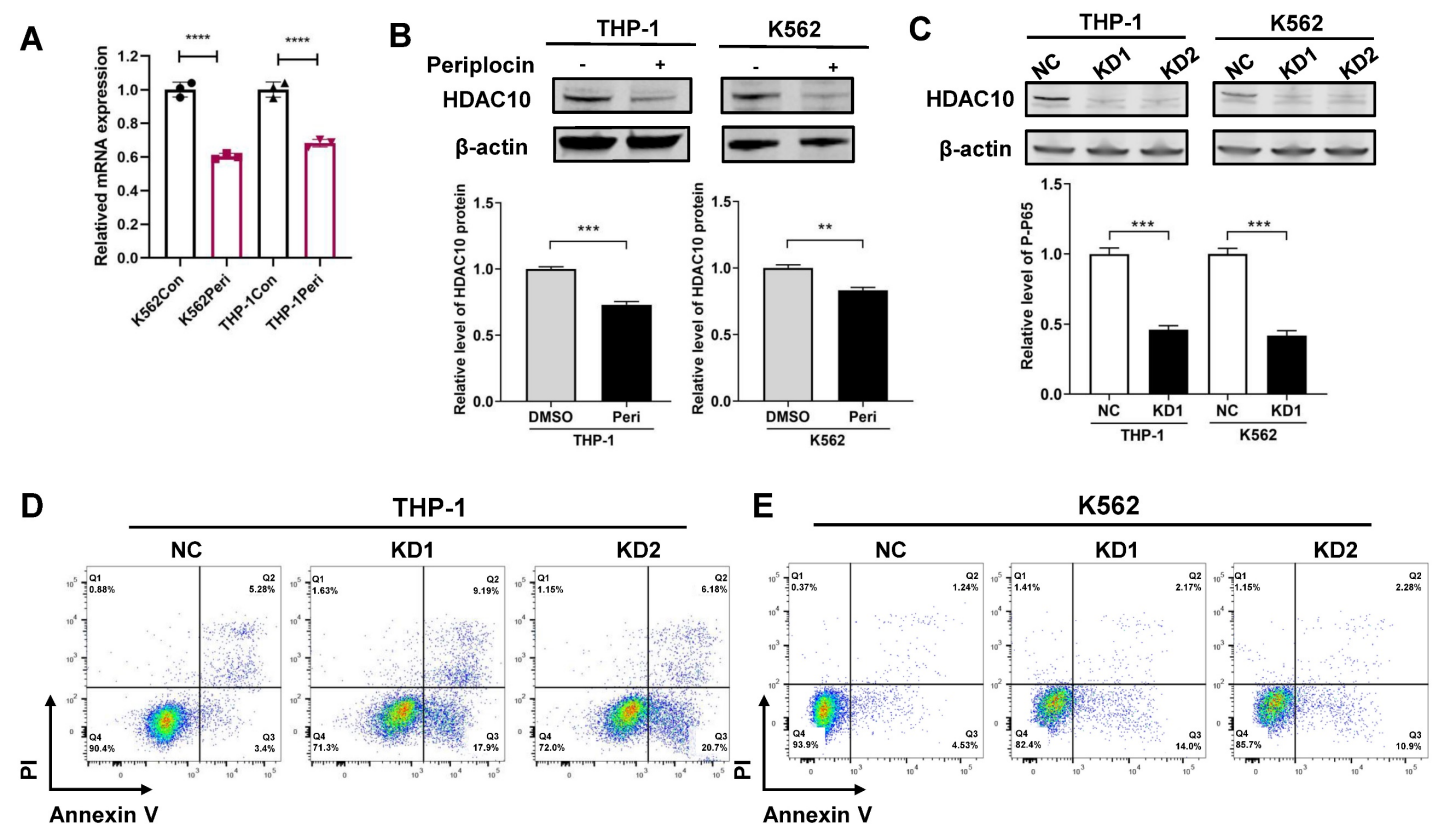
** Periplocin downregulates HDAC10 expression and promotes apoptosis in leukemia cells.** (A) Quantitative real-time PCR analysis of HDAC10 mRNA levels in K562 and THP-1 cells treated with DMSO (control) or Periplocin (Peri). (B) Western blot analysis of HDAC10 protein expression in K562 and THP-1 cells treated with DMSO or Periplocin. β-actin served as a loading control. Quantified protein levels are shown in the bar graphs. (C) Western blot validation of HDAC10 knockdown efficiency in two independent shRNA knockdown clones (KD1 and KD2) in K562 and THP-1 cells. β-actin was used as a loading control. Quantified protein levels are shown in the bar graphs. (D) Representative flow cytometry plots of Annexin V/PI staining in HDAC10 knockdown (KD1 and KD2) and negative control (NC) THP-1 cells. (E) Representative flow cytometry plots of Annexin V/PI staining in HDAC10 knockdown (KD1 and KD2) and negative control (NC) K562 cells. Data are presented as mean ± SD. **p < 0.01; ***p < 0.001; ****p < 0.0001.

To better understand the mechanism, we performed fundamental experiments to validate how Periplocin influences HDAC10 function and activity. In line with prior data analysis, quantitative PCR analysis showed a significant decrease in HDAC10 mRNA levels following Periplocin treatment, with a reduction of approximately 30-40% observed in both K562 and THP-1 cells compared to untreated control (**Figure 6A**). Western blot analysis confirmed a corresponding decrease in HDAC10 protein levels, underscoring a robust and consistent response across these two cell lines (**Figure 6B**). By validating HDAC10 expression at both the transcriptional and protein levels, we propose that HDAC10 is a key target through which Periplocin exerts its anti-leukemia effects. To verify the functional relevance of HDAC10 as a potential target, we generated two independent HDAC10 knockdown cell lines (KD1 and KD2) in both THP-1 and K562 cells. Western blot analysis confirmed effective silencing of HDAC10 expression in both cell lines (**Figure 6C**). Apoptosis was subsequently assessed by Annexin V/PI double staining and flow cytometry. Compared to the negative control group, HDAC10 knockdown led to a significant increase in apoptotic cells in both THP-1 (**Figure 6D**) and K562 (**Figure 6E**). This phenotype closely mirrors the pro-apoptotic effect observed following Periplocin treatment, suggesting that HDAC10 downregulation is a key mechanism contributing to Periplocin-induced apoptosis in leukemia cells.

**Figure 7:**
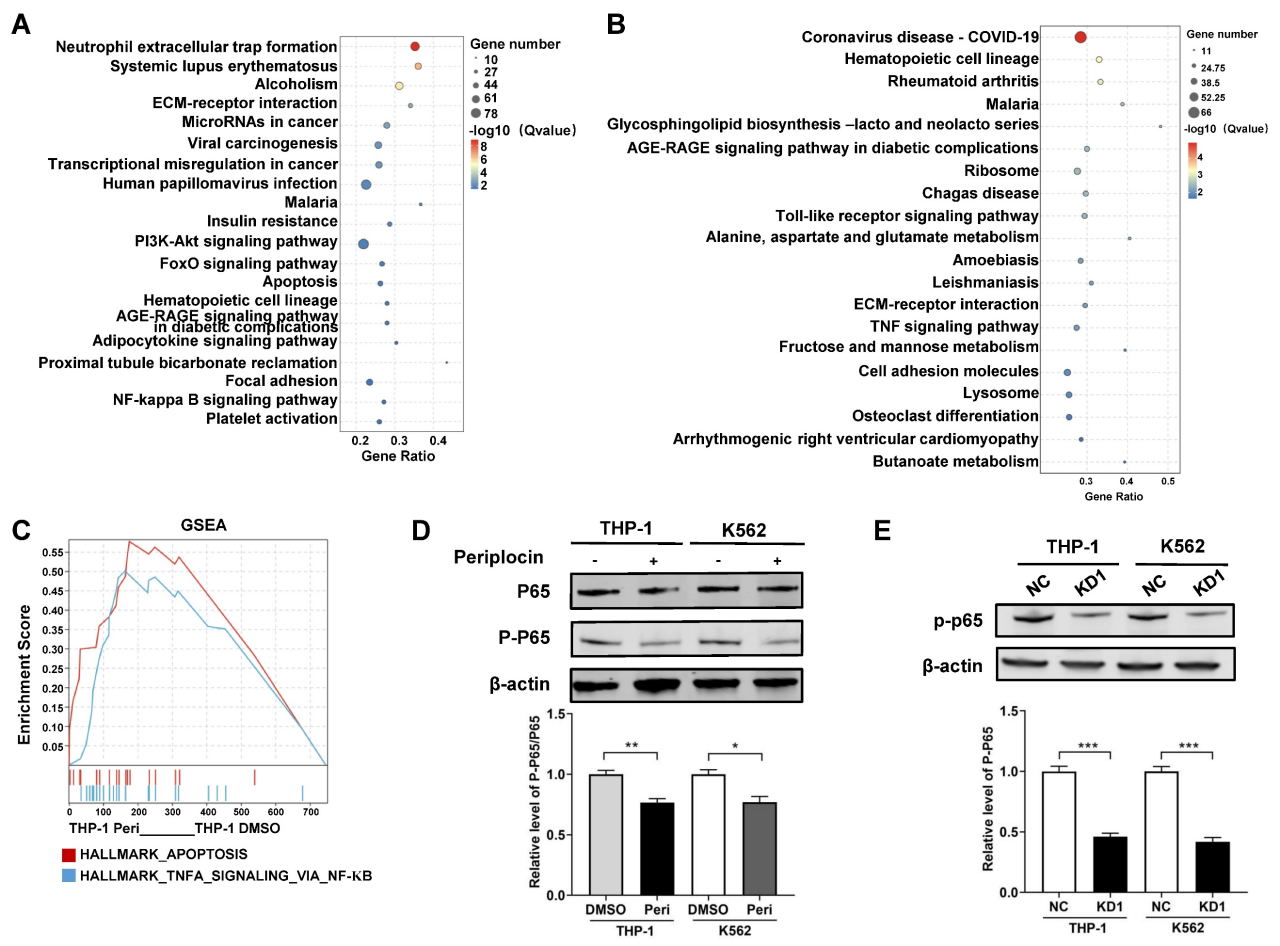
**Periplocin suppresses NF-κB signaling through inhibition of HDAC10.** (A-B) KEGG pathway enrichment analysis of DEGs between Periplocin-treated and DMSO-treated K562 (A) and THP-1 (B) cells. (C) GSEA enrichment analysis of Periplocin-treated THP-1 cells. (D) Western blot analysis of total p65 and phosphorylated p65 in THP-1 and K562 cells treated with DMSO or Periplocin. β-actin served as a loading control. (E) Western blot analysis of P-P65 expression in HDAC10 knockdown cells (KD1) and negative control (NC) in THP-1 and K562 cell lines. Data are presented as mean ± SD. *p < 0.05; **p < 0.01; ***p < 0.001.

Based on the RNA-seq results, we analyzed pathway enrichment in K562 and THP-1 cells separately to explore downstream pathways of HDAC10. Both cell lines showed distinct pathway profiles, with K562 significantly enriched in the NF-κB and PI3K-Akt pathways (**Figure 7A**), while THP-1 was enriched in the TNF signaling pathway (**Figure 7B**). This was particularly intriguing, as the TNF signaling pathway is well-documented to interact with NF-κB, a key regulator of apoptosis and inflammation. To further investigate this interaction, we performed GSEA on THP-1 cells, which revealed Tumor necrosis factor-alpha (TNFA) signaling via NF-κB as a critical pathway (**Figure 7C**). These results point to NF-κB as a shared downstream mediator of Periplocin's effects, potentially linking its actions across different leukemia subtypes. Western blot analysis showed a marked decrease in P-P65 levels in Periplocin-treated cells, whereas the phosphorylation level of AKT remained unchanged, suggesting inhibition of NF-κB activation (**Figure 7D**, **Supplementary Figure 1B**). To further clarify the relationship between Periplocin, HDAC10, and P-P65, we examined P-P65 expression in HDAC10 knockdown leukemia cell lines. Similar to the effect of Periplocin treatment, HDAC10 knockdown resulted in a significant reduction in P-P65 levels in both THP-1 and K562 cells (**Figure 7E**). These results indicate that Periplocin may induce apoptosis in leukemia cells partially through HDAC10 inhibition, which appears to be associated with downregulation of NF-κB activity.

## 4. Discussion

HDAC10, a member of the class IIb HDAC family, is crucial for regulating chromatin accessibility through its deacetylase activity, consistent with its pivotal role in chromatin remodeling and gene expression control 16. In our research, we first confirmed the tumor-suppressive effects of periplocin in myeloid leukemia that it can effectively induce apoptosis and cell cycle arrest, highlighting its therapeutic potential. Through transcriptomic sequencing and network pharmacology analyses, HDAC10 was identified as a key downstream target and our experiments further validated that periplocin significantly reduced HDAC10 expression levels (**Figures 6-7**). Taken together, these data suggest that periplocin exerts its antitumor effects, at least in part, by downregulating HDAC10.

HDAC10 plays a multifaceted role in cancer progression, contributing to drug resistance, cell cycle regulation, and immune evasion. For instance, Feng *et al.* demonstrated that HDAC10 deacetylates YAP1—a tumor suppressor protein in FLT3-ITD+ AML—resulting in its inactivation and promoting leukemia drug resistance 34. The HDAC inhibitor chidamide effectively reversed this resistance by restoring YAP1 activity. Similarly, in melanoma, HDAC10 inhibitors were shown to enhance tumor sensitivity to BRAF inhibitors by upregulating SPARC expression, a key factor in modulating the tumor microenvironment 35. In non-small cell lung cancer (NSCLC), HDAC10 was found to regulate the G2/M phase transition through cyclin A expression, underscoring its critical role in cell cycle control 36. Beyond its roles in tumor cell survival and proliferation, HDAC10 also contributes to immune evasion. For example, it modulates immune checkpoint molecules such as PD-L1, enabling tumor cells to evade immune surveillance 37. Furthermore, HDAC inhibitors have been reported to disrupt the tumor microenvironment by suppressing the activity and aggregation of myeloid-derived suppressor cells (MDSCs), thereby enhancing tumor-specific immune responses 38.

We hypothesize that Periplocin exerts its biological effects by specifically targeting the active site of HDAC10 through molecular docking analysis. Periplocin was found to form stable interactions within HDAC10's active pocket, including hydrophobic interactions with ALA629 and hydrogen bonds with residues such as LEU118, GLN123, GLU628, ASN626, ASP428, and MET1. These interactions not only anchor Periplocin within the active site of HDAC10 but also likely inhibit its catalytic activity, disrupting downstream epigenetic regulatory pathway, which may contribute to tumor suppression by halting cell growth and inducing apoptosis.

Notably, by binding to the highly conserved active hydrophobic pocket of HDAC10, periplocin could maintain a certain level of drug specificity and high binding affinity, which provide valuable structural insights into the design of HDAC10-specific therapies with potential applications in leukemia treatment.

One of the downstream pathways examined in our study is the NF-κB signaling pathway. While histones are the most well-characterized substrates of HDACs, increasing evidence reveals that HDACs also target non-histone proteins, expanding their regulatory roles. For instance, in the NF-κB pathway, acetylated RelA (p65) can be deacetylated by HDAC3, which facilitates its interaction with IκBα and subsequently terminates NF-κB signaling 16, 39-40. Based on our RNA-seq and pathway enrichment results, both K562 and THP-1 cells exhibited activation of the NF-κB signaling pathway following Periplocin treatment. However, the upstream regulatory mechanisms appear to differ between these leukemia subtypes. In THP-1 cells, KEGG and GSEA analyses suggest that TNFA may act as a key upstream regulator that mediates NF-κB activation.

TNFA is a well-characterized pro-inflammatory cytokine known to activate the NF-κB pathway through its interaction with TNF receptors (TNFR1 and TNFR2) 41. Upon binding to TNFR1, TNFA initiates a cascade involving adaptor proteins such as TRADD, TRAF2, and RIPK1, leading to the activation of the IκB kinase (IKK) complex 41-42. This, in turn, phosphorylates IκBα, resulting in its degradation and subsequent translocation of NF-κB (p65/p50) into the nucleus, where it regulates target gene expression involved in inflammation, proliferation, and apoptosis resistance 43.

These mechanisms are likely context-dependent and may vary across cell types and pathological conditions. In our study, treatment with Periplocin significantly reduced HDAC10 levels in both K562 and THP-1 cell lines, along with decreased expression of both total p65 and p-p65, suggesting a potential regulatory link between HDAC10 and NF-κB signaling. But the precise mechanism-whether through direct interaction with NF-κB components or via upstream kinases-requires further investigation.

## 5. Conclusion

Our study highlights the therapeutic potential of Periplocin in myeloid leukemia, where it effectively suppresses tumor growth by inducing apoptosis and enforcing cell cycle arrest. Through transcriptomic sequencing and network pharmacology analyses, we identified HDAC10 as a critical downstream target of Periplocin. The compound successfully reduced HDAC10 expression, leading to the inhibition of NF-κB pathway activity. These findings provide a solid foundation for exploring Periplocin as a novel HDAC-targeted agent, with promising applications in enhancing leukemia treatment strategies.

## Supplementary Material

Supplementary figure.

Supplementary table 1.

## Abbreviations

AML: Acute Myeloid Leukemia
HSCs: Hematopoietic Stem Cells
LSCs: Leukemia Stem Cells
m6A: N6-Methyladenosine
HDAC: Histone Deacetylase
HDACi: Histone Deacetylase Inhibitor
TNFA: Tumor Necrosis Factor Alpha
NF-κB: Nuclear Factor-kappa B
PI3K-AKT: Phosphoinositide 3-kinase/Protein Kinase B Pathway
