# Supplementary material for: Periplocin Targets HDAC10 to Inhibit NF-κB Signaling and Induce Apoptosis in Myeloid Leukemia Cells
Source: J Cancer. 2025 Jun 23;16(9):2970–83. doi: 10.7150/jca.113591 (PMC12244337; doi:10.7150/jca.113591)
Supplement: Supplementary file 1 — Supplementary figure. [file jcav16p2970s1.pdf]

Supplementary Figure 1. (A) HDAC10 expression across normal hematopoietic stem and progenitor cells (HSPCs) and various leukemia subtypes, based on BloodSpot database analysis. (B) WB analysis of phosphorylated Akt (p-Akt) levels in THP-1 and K562 cells following Periplocin treatment (100 nM, 72 h).

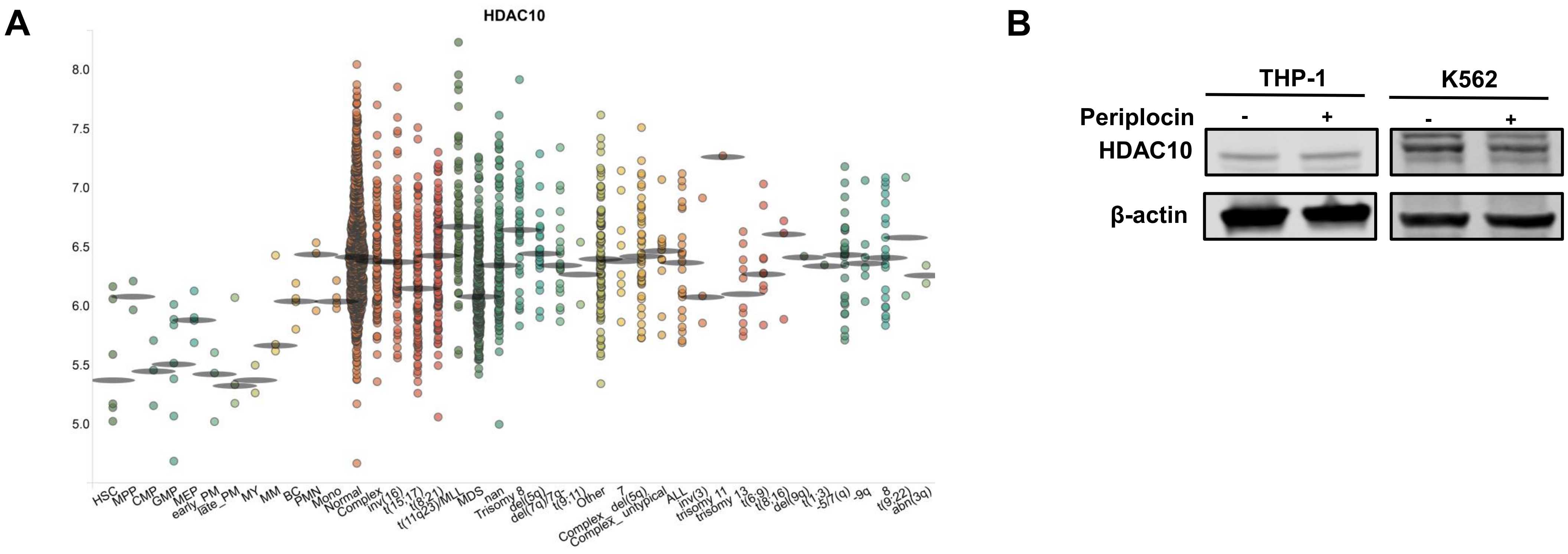
A
HDAC10
8.0
7.5
7.0
6.5
6.0
5.5
5.0
HSC
MPP
CMP
GMP
MEP
early_PM
late_PM
MY
MM
BC
PMN
Mono
Normal
Complex
inv(16)
t(15;17)
t(8;21)
t(11q23)/MLL
MDS
nan
Trisomy 8
del(5q)
del(7q)/7q-
t(9;11)
Other
ALL
B
THP-1
K562
Periplocin
-
+
-
+
HDAC10
β-actin
